# Parameter-Efficient Audio-Visual Dynamic Facial Expression Recognition with Mamba Fusion Adapters and Frame-Level Feature Arrangement

**DOI:** 10.3390/s26175384

**Published:** 2026-08-26

**Authors:** Kangbo Ning, Shanshan Gao, Zhaoqiang Xia, Dong Huang, Lei Li

**Affiliations:** 1 School of Biomedical Engineering, Air Force Medical University, Xi’an 710032, China; 232115029@stu.sdufe.edu.cn; 2 Shaanxi Provincial Key Laboratory of Bioelectromagnetic Detection and Intelligent Perception, No. 169 Changle West Road, Xi’an 710032, China; 3 School of Computing and Artificial Intelligence, Shandong University of Finance and Economics, Jinan 250014, China; gss_sdufe@sdufe.edu.cn; 4 School of Electronics and Information, Northwestern Polytechnical University, Xi’an 710072, China; zxia@nwpu.edu.cn

**Keywords:** dynamic facial expression recognition, audio-visual fusion, parameter-efficient fine-tuning, Mamba fusion adapter, frame-level feature arrangement (FFA)

## Abstract

Dynamic Facial Expression Recognition (DFER) has recently attracted significant interest due to its vital role in enabling empathetic and human-compatible technologies. Developing models that remain robust under in-the-wild variability is a key motivation for DFER research and its practical applications. Improving models through multimodal learning, which leverages audio and video data for richer, complementary representations, is one promising direction. However, existing methods still rely heavily on modality-specific encoders and coarse-grained content-level alignment, which hinders their ability to capture fine-grained emotional semantics and dynamic cross-modal interactions. To address this, we adopt parameter-efficient fine-tuning (PEFT) to facilitate audio-visual interaction. This strategy offers key advantages: (1) freezing parameters preserves upstream pretrained knowledge, ensuring that the model focuses solely on learning modules for audio-visual interaction and modal fusion; (2) a Mamba Fusion Adapter (MFAdapter) is inserted at each encoder layer to perform causal, audio-conditioned fusion over a frame-aligned token sequence, enabling efficient multi-level cross-modal injection with linear complexity; and (3) a Frame-level Feature Arrangement (FFA) strategy is introduced as a deterministic index-based arrangement scheme that arranges audio tokens at the video-frame rate, providing a frame-indexed temporal prior that supports the causal scan in MFAdapter; FFA reduces, but does not eliminate, coarse segment-level mismatch and is not claimed as verified frame-level synchronization. Notably, our method achieves competitive performance on DFEW and MAFW while updating 2.7% (4.7 million) of the model parameters, with the best WAR of 58.70% on MAFW among all compared methods and 76.62%/65.25% (WAR/UAR) on DFEW under the official five-fold cross-validation protocols.

## 1. Introduction

Affective computing (AC) has rapidly evolved into an influential research field, with many countries prioritizing the integration of Artificial Intelligence (AI) and AC as a strategic frontier [1]. Within this field, Facial Expression Recognition (FER) plays an important role in analyzing observable affective behavior and supporting applications such as psychological research, affect-aware human–computer interaction, and behavioral analysis [2,3,4]. However, the inherent ambiguity of facial cues alone makes it difficult to reliably infer emotional states without supporting contextual information. Accordingly, this task should be framed as the recognition of apparent, expressed affect rather than the inference of private subjective experience.

To address these limitations, Dynamic Facial Expression Recognition (DFER) leverages video sequences to model the temporal progression of expressions [1,5]. Early research on DFER relied on laboratory-controlled datasets [6], where expressions followed predictable patterns with uniform intensity, thus failing to capture real-world complexity. The introduction of in-the-wild datasets such as DFEW [7] and MAFW [8] significantly advanced the field by capturing spontaneous expressions across diverse scenes [9,10,11]. Nevertheless, the high cost and difficulty of annotation restrict dataset scale and limit generalization to open-world conditions. Furthermore, video-only DFER methods struggle under adverse conditions, such as low illumination, occlusion, or motion blur, where subtle cues, such as eye or mouth movements, are easily missed. Concurrent progress on the modeling side has continued to push the frontier of DFER: for example, recent efficient self-supervised video backbones such as AdaTosk [12] introduce adaptive temporal soft masking to suppress semantic redundancy and reduce computational cost, while cross-modal alignment frameworks such as GRACE [13] align fine-grained linguistic cues with visually salient regions via entropy-regularized optimal transport, both reporting state-of-the-art performance on DFEW, FERV39k, and MAFW. These works illustrate the field’s rapid transition from coarse, segment-level supervision to fine-grained, frame- and token-level reasoning, which further motivates a temporally precise and parameter-efficient multimodal DFER design. Without complementary modalities, these models may have difficulty integrating potentially informative vocal context; however, neither unimodal nor multimodal observations provide direct access to a person’s genuine internal emotional state. Accordingly, the task should be framed as the recognition of apparent, expressed affect rather than the inference of private subjective experience.

To enhance robustness and semantic understanding, recent research has shifted toward multimodal fusion, where audio cues complement visual signals [14,15]. Audio prosodic features, for instance, can reinforce visual cues—such as a cheerful tone supporting the recognition of a smile or a low-pitched voice indicating sadness. Despite these benefits, current audio–visual DFER methods remain limited by several key challenges. The conventional pretraining–fine-tuning paradigm may overwrite modality-specific representations when fine-tuning data are scarce, since the optimization budget must be shared with task-specific objectives [16,17,18]; modality-specific knowledge, such as visual facial representations or prosodic sensitivity, can therefore be diluted by the joint loss. Moreover, most existing fusion mechanisms fail to effectively capture dynamic intermodal interactions, relying on simple concatenation or temporal transformers that cannot model fine-grained synchrony, such as the correspondence between a frown and an angry tone. In addition, feature alignment in current approaches is typically coarse-grained, performed at the segment or layer level [19], which results in temporal mismatches, such as pairing a neutral frame with a cheerful vocal cue.

To address the above challenges, this work proposes a lightweight multimodal fusion architecture for DFER, integrating parameter-efficient fine-tuning (PEFT) with the efficient sequential modeling capability of Mamba. PEFT preserves modality-specific representations by freezing pretrained audio and visual encoders, so that the optimization budget is concentrated on cross-modal fusion rather than on re-fitting the encoders to a small target corpus. To enable efficient audio-conditioned visual adaptation, we introduce the Mamba Fusion Adapter (MFAdapter), which is inserted between corresponding layers of the two encoders. MFAdapter concatenates frame-aligned audio and video tokens in a fixed order and applies a standard causal selective scan, so that visual tokens can absorb preceding acoustic context with linear complexity. Furthermore, to overcome coarse feature alignment, we design a Frame-level Feature Arrangement (FFA) strategy that imposes a deterministic, index-based correspondence between audio tokens and video frames. Rather than estimating or verifying audio–visual synchrony, FFA provides a structured temporal prior that is compatible with the subsequent causal scan of MFAdapter; we therefore refer to it as imposing a frame-indexed ordering rather than eliminating temporal misalignment. Together, FFA provides the temporal structure required for ordered scanning, while MFAdapter injects cross-modal cues hierarchically into the frozen encoders; complementary late fusion in the temporal classifier further aggregates multimodal evidence for recognition. The main contributions can be summarized as follows:

- We propose a parameter-efficient audio-visual DFER framework that leverages PEFT for adaptive multimodal fusion. By freezing pretrained encoders and training only cross-modal interaction modules, our method maximizes the utilization of modality-specific prior knowledge. The frozen-backbone design also preserves the representations learned by the large-scale self-supervised AudioMAE and MAE-Face encoders and concentrates the limited optimization budget on the cross-modal fusion pathway, which together act as an implicit regularizer against majority-class overfitting on small target corpora such as DFEW.
- We develop the Mamba Fusion Adapter (MFAdapter), which performs causal selective scanning over a concatenated audio-visual token sequence. By placing frame-aligned audio tokens as a prefix to video tokens, MFAdapter realizes efficient audio-conditioned cross-modal injection at multiple encoder depths, rather than assuming bidirectional or symmetric fusion within a single scan.
- We propose Frame-level Feature Arrangement, a deterministic index-based arrangement strategy that explicitly structures audio tokens at the video-frame rate. This structuring provides a frame-indexed temporal prior that supports the ordered causal scan in MFAdapter and reduces, but does not eliminate, temporal mismatch between audio patches and video frames at the resolution of 4 audio patches spanning 640 ms per video frame; we treat the reduction of coarser segment-level mismatch as one benefit among several rather than as a guarantee of true audio–visual synchronization.
- We validate the effectiveness of our approach on two challenging in-the-wild DFER benchmarks, DFEW and MAFW. Under the official five-fold cross-validation protocols, our method achieves competitive performance on DFEW and MAFW while updating 2.7% (4.7 M) of the model parameters (76.62% WAR/65.25% UAR on DFEW; 58.70% WAR/43.66% UAR on MAFW, where 58.70% WAR is the highest among the compared methods and 43.66% UAR is the second highest). This demonstrates the parameter efficiency of our framework and its competitive performance under the official within-dataset five-fold cross-validation protocols of DFEW and MAFW.

## 2. Related Work

### 2.1. Dynamic Facial Expression Recognition

In previous studies, DFER methods have evolved from handcrafted features in controlled settings to deep learning approaches that can handle in-the-wild variability through large-scale datasets.

#### 2.1.1. Unimodal DFER Methods

Recent advances in Dynamic Facial Expression Recognition (DFER) have evolved from handcrafted feature-based methods in controlled laboratory settings to deep learning approaches capable of handling in-the-wild variability through large-scale datasets. Current methodologies are broadly classified into three paradigms: (1) Hybrid 2D CNN-RNN architectures [20,21] that decouple spatial-temporal modeling, exemplified by Wang et al.’s DPCNet [21] which extracts keyframe features via spatial-frame excitation and channel-temporal aggregation modules; (2) 3D CNNs [7,22] for joint spatiotemporal feature extraction at increased computational cost; and (3) Transformer-based frameworks, such as Xia et al.’s multi-scale spatial-temporal aggregator [23] and models like FormerDFER [11] that leverage attention for unified fusion. Innovations within these categories include Liu et al.’s CEFLNet [24], combining self-attention with local-global relation learning for emotional intensity recognition, Li et al.’s GCA block, refining SE mechanisms with intensity-aware loss [25], Tao et al.’s interpretable Freq-HD for dynamic clip selection [26], and Kawamura et al.’s MIDAS approach, using probabilistic soft labels to enhance ambiguous expression modeling [27]. Recently, Chen et al. proposed CFAN-SDA [28], which introduces a coarse-to-fine learning strategy combining static-to-dynamic adaptation and hierarchical spatial-temporal transformers to improve robustness against noisy frames, pose variations, and occlusions. In parallel, AdaTosk [12] couples supervised classification with self-supervised reconstruction through an adaptive temporal soft mask that dynamically adjusts masking strength across time, achieving a 3% improvement with 10M fewer parameters and 6G fewer FLOPs on FERV39K, DFEW, and MAFW. Its adaptive token-level masking strategy motivates our extension to multimodal frame-level design. This progression demonstrates the field’s maturation toward architectures that balance temporal modeling precision, representational richness, and real-world applicability.

#### 2.1.2. Multimodal DFER Methods

Recent progress in multimodal learning [14,29] has shown that integrating complementary modalities improves the robustness of emotion recognition. Several multimodal DFER methods exemplify this trend: FineCLIPER [30] aligns visual and language representations but lacks explicit temporal modeling, and VAEmo [31] introduces a unified encoder with a two-stage pretraining paradigm that first learns general audio–visual correspondence and then injects emotion-aware knowledge through dual-path contrastive learning. PFFEI [32] performs parameter-free feature evaluation and joint interaction, using the normalization scaling factor as a feature-quality indicator to adaptively regulate cross-modal fusion and alleviate modality imbalance under noisy in-the-wild conditions, all without adding trainable parameters. Within the same period, PEFT-based DFER frameworks have emerged as a parallel branch: MMA-DFER [33] freezes both encoders and injects per-layer Fusion Bottlenecks that project audio to dim = 128 and modulate both modalities via mean-pooled additive fusion (O(*L*)) injected as residual at each layer, with three learnable prompts inserted every six layers. Unlike this pooled-additive modulation, our MFAdapter performs causal selective scanning (O(*L*)) over frame-aligned token sequences, achieving fine-grained token-level audio-conditioned visual adaptation. GRACE [13] recently demonstrates that token-level cross-modal alignment—via coarse-to-fine affective text enhancement and motion-difference weighting—achieves strong results on DFEW, FERV39k, and MAFW. We note that several concurrent works report higher absolute metrics on DFEW; we position our method as competitive among PEFT-based approaches with strict parameter constraints, achieving the best WAR on MAFW (58.70%) among all compared methods. This motivates our frame-indexed grouping for token-level cross-modal interaction, though we adopt a different modality pair and fusion mechanism.

Although recent advances have substantially improved multimodal DFER, most approaches remain limited by insufficient temporal granularity and rigid fusion mechanisms. Single-modal models struggle to capture cross-frame emotional dynamics, while existing multimodal methods, such as FineCLIPER [30], VAEmo [31], and PFFEI [32], either emphasize global semantic alignment or focus on feature quality assessment rather than fine-grained temporal reasoning. These constraints hinder the modeling of subtle, continuous emotional transitions that emerge from complex audio-visual interactions. To overcome these challenges, we propose an audio-visual multimodal fusion framework that couples frame-indexed temporal structuring with causal, audio-conditioned cross-modal injection via the MFAdapter, enabling structured temporal grouping for the fusion module and efficient multimodal representation learning. Beyond facial cues, H2OFormer [34] also targets fine-grained affective cues via joint self-supervised reconstruction and supervised learning, but operates on skeleton sequences using hypergraph attention. In contrast, our MFAdapter handles audio-visual modalities through causal Mamba-based cross-modal scanning, offering a complementary approach to fine-grained emotion modeling. Outside DFER, MambaFusion [35] demonstrates the viability of SSM-based multimodal fusion for autonomous driving, but relies on bidirectional cross-attention and spatial reliability gating, which incur higher complexity. Our MFAdapter instead leverages the inherent sequential bias of causal Mamba for efficient audio-conditioned visual adaptation, making it particularly suitable for resource-constrained DFER scenarios.

### 2.2. Parameter-Efficient Fine-Tuning

Parameter-efficient fine-tuning (PEFT) is an effective technique for transferring large pretrained models to downstream tasks and has been extensively studied. It efficiently addresses the high cost of full-parameter fine-tuning and has shown significant success in alleviating catastrophic forgetting. PEFT methods are mainly categorized into three types: (1) Adapter-based methods [36], which fine-tune the model by inserting adapter modules without adjusting all model parameters; (2) Prompt-tuning methods [37], which adapt the model to specific tasks by optimizing input prompts while keeping model parameters unchanged; and (3) LoRA-based methods [38,39], which employ low-rank matrix decomposition techniques to train only the low-rank matrix parameters, effectively reducing computational and memory overhead. Although these methods have been widely applied, this work primarily focuses on audio-visual interaction. Compared with the other two methods, the adapter paradigm is more suitable for facilitating modality interaction, as it inserts lightweight sub-modules between encoder layers, enabling efficient cross-modal information fusion.

Although these approaches have achieved notable success, most prior research has focused on unimodal tasks, with only limited exploration of multimodal settings, such as video–text learning in vision–language models [14,40]. In contrast, DFER presents unique challenges: audio-visual inputs require fine-grained temporal alignment, robust cross-modal interaction, and resilience to noise or data imbalance. Full-parameter tuning of multimodal encoders not only incurs substantial training cost but also risks overwriting modality-specific knowledge that is crucial for robust representation learning. To address these issues, we adopt an adapter-based PEFT paradigm, which is particularly well suited for multimodal DFER. By inserting lightweight adapters into each Transformer block, our method preserves pretrained unimodal knowledge while directing the learning capacity toward cross-modal interaction, thereby enhancing temporal sensitivity and improving recognition performance with only a minimal number of trainable parameters. Recent work also examines the synergy between PEFT and Mamba backbones: Yoshimura et al. [41] demonstrate that SSMs benefit more from parameter-efficient adaptation than Transformers, supporting our choice of combining PEFT with Mamba. In contrast to their general-purpose benchmarking, we apply PEFT–Mamba to the audio–visual DFER task, where the unidirectional causal scan of MFAdapter is deliberately designed to propagate audio cues as global conditioning context into visual tokens.

## 3. Proposed Method

**Figure 1:**
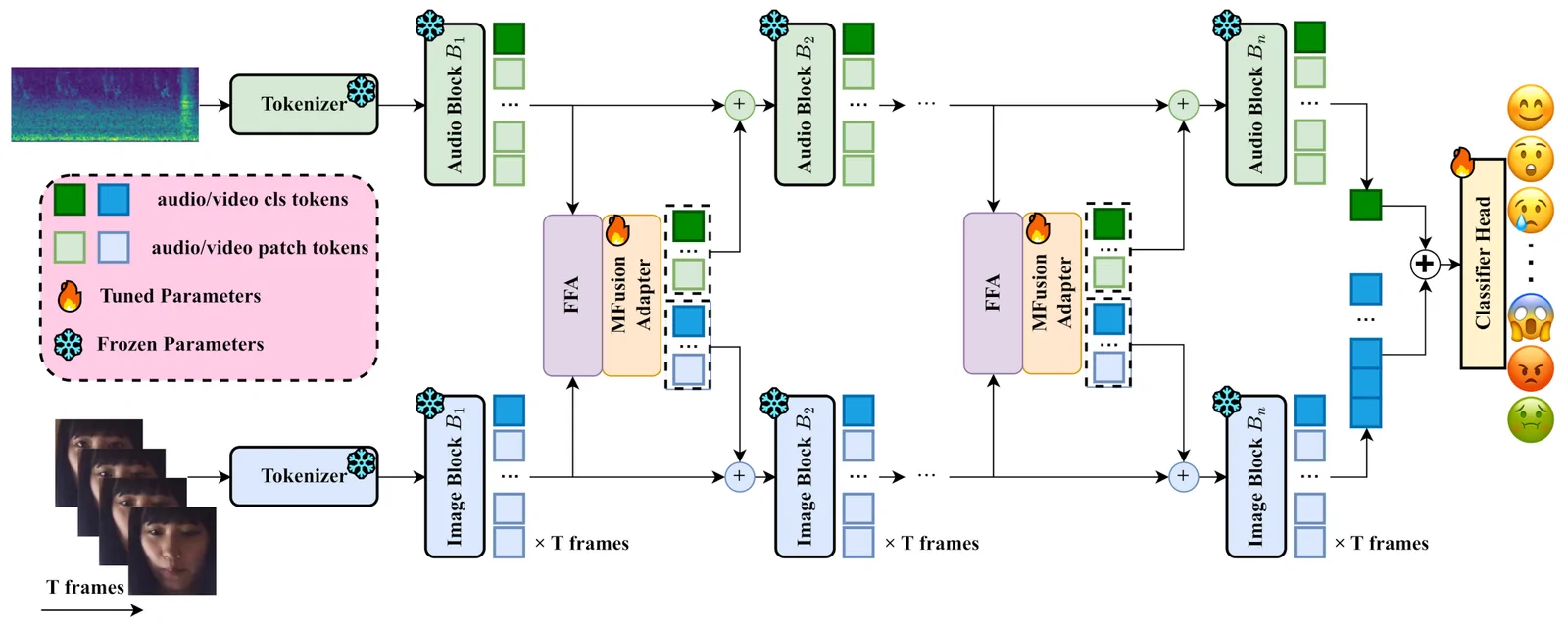
Overview of the proposed framework. FFA implements a frame-indexed token-grouping strategy that organizes audio tokens at the video-frame rate, and MFAdapter inserts a causal Mamba fusion block after each paired encoder layer. Within each MFAdapter, frame-indexed audio tokens are placed before video tokens and scanned unidirectionally, enabling audio-conditioned visual adaptation at multiple feature levels.

This section presents an overview of our proposed framework, illustrated in Figure 1. Our approach is designed to learn robust emotion representations from the vast amounts of video and audio data available online, leveraging the complementary affective cues inherent in each modality. At the core of our framework is a simple yet effective adapter-based architecture combined with cross-modal fusion, which enables efficient multimodal learning.

We begin by revisiting the preliminaries of State Space Models (SSMs) as a theoretical foundation. We then present FFA and MFAdapter, with explicit discussion of token ordering and the causal scan used for audio-conditioned fusion. Next, we describe the backbone architectures selected for the audio and visual modalities. Finally, we introduce the temporal classifier head that aggregates multimodal cues for prediction.

### 3.1. Preliminaries

We review SSMs in three clearly separated stages: the classical continuous linear time-invariant (LTI) formulation; its discretization and the convolution representation that holds only for discrete LTI systems; and the selective, input-dependent SSM used by Mamba, which is time-varying and therefore computed by a recurrent selective scan rather than by a fixed convolution kernel. Throughout, the input is denoted by *x* (continuous x(t) or discrete $x_{t}$).

**Classical Continuous LTI SSM.** State-space models are widely used to map an input signal x(t)∈R to an output y(t)∈R through a latent state $h\left(t\right)\in R^{N}$. In the classical continuous LTI setting, the dynamics are governed by linear ordinary differential equations with time-invariant parameters:

(1) $$h^{\prime }\left(t\right)=Ah(t)+Bx(t),$$

(2) y(t)=Ch(t)+Dx(t),

where $h^{\prime }\left(t\right)$ is the time derivative of the hidden state, $A\in R^{N\times N}$ is the evolution matrix, and $B\in R^{N\times 1}$, $C\in R^{1\times N}$, D∈R are projection parameters. Importantly, (A,B,C,D) do not depend on *t*.

**Discretization and LTI Convolution Form.** To implement SSMs in deep learning frameworks, the continuous system is discretized. The Zero-Order Hold (ZOH) [42] is commonly used for LTI systems: for a step size Δ, it maps (A,B,C,D) to discrete parameters $\left(\bar{A},\bar{B},\bar{C},\bar{D}\right)$ as follows:

(3) $$\begin{array}{cc} \bar{A} & =\exp(\Delta A), \end{array}$$

(4) $$\begin{array}{cc} \bar{B} & ={\left(\Delta A\right)}^{-1}\left(\exp(\Delta A)-I\right)\cdot \Delta B, \end{array}$$

(5) $$\begin{array}{cc} \bar{C} & =C,\;\bar{D}=D. \end{array}$$

When these discrete parameters remain time-invariant, the recurrence admits an equivalent global convolution with a structured kernel $\bar{K}\in R^{L}$ for an input sequence $x=(x_{1},\ldots ,x_{L})$:

(6) $$\bar{K}\;=\left(\bar{C}\;\bar{B},\;\bar{C}\;\bar{A}\;\bar{B},\;\ldots ,\;\bar{C}\;{\bar{A}}^{L-1}\bar{B}\right),$$

(7) $$y=x*\bar{K}.$$

Equations (6) and (7) characterize discrete LTI SSMs only. They do *not* describe the selective Mamba model introduced next, whose parameters vary with the input and therefore preclude a single time-invariant convolutional kernel. From this point on, the LTI convolution form is abandoned entirely; the selective model uses only the recurrent form given below.

**Selective SSM Used in Mamba.** To overcome the limited context selectivity of LTI SSMs, Mamba [43] introduces a selection mechanism (S6) that generates input-dependent parameters ${\left\{B_{t},C_{t},\Delta_{t}\right\}}_{t=1}^{L}$ from the sequence. After discretization, the effective transition and projection parameters become time-varying, denoted $\left({\bar{A}}_{t},{\bar{B}}_{t},C_{t}\right)$. Because the system is no longer LTI, the fixed-kernel convolution in Equations (6) and (7) does not apply. Instead, hidden states are computed by a causal recurrent selective scan:

(8) $$\begin{array}{cc} h_{t} & ={\bar{A}}_{t}\;h_{t-1}+{\bar{B}}_{t}\;x_{t}, \end{array}$$

(9) $$\begin{array}{cc} y_{t} & =C_{t}\;h_{t}+D\;x_{t}. \end{array}$$

Here, the hidden state $h_{t}$ is computed by the discrete-time recurrence in Equation (8), which consumes the previous state $h_{t-1}$ together with the current input $x_{t}$ through the time-varying parameters $\left({\bar{A}}_{t},{\bar{B}}_{t}\right)$; the output at step *t* is then given by Equation (9), which projects $h_{t}$ back to the output space via $C_{t}$. In our MFAdapter, ${\mathrm{Mamba}}_{\mathrm{causal}}$ refers exclusively to this selective scan along an ordered token sequence, rather than to the LTI convolution form in Equations (6) and (7).

### 3.2. Visual Encoder and Audio Encoder

Our approach leverages two publicly available, off-the-shelf unimodal encoders that have been fine-tuned via self-supervised learning (SSL) for the audio and visual modalities. These encoders are independently pretrained on audio sequences and static facial images, respectively, and are later adapted to handle dynamic audio-visual facial expression recognition in unconstrained, real-world environments.

Specifically, we select models pretrained with the Masked Autoencoder (MAE) reconstruction objective because of MAE’s proven effectiveness in capturing fine-grained features for downstream tasks [44]. As backbone encoders, we employ two publicly available ViT-based models: AudioMAE [45] for audio inputs and MAE-Face [46] for visual inputs. Both models adopt the standard ViT-Base [47] configuration with 12 Transformer encoder layers, ensuring consistent network depth. AudioMAE is pretrained on the large-scale AudioSet dataset [48], which covers diverse audio events. In contrast, MAE-Face is pretrained on static facial images from the AffectNet dataset, a large-scale, in-the-wild facial expression database specifically designed for emotion recognition tasks.

### 3.3. Frame-Level Feature Arrangement (FFA)

In multimodal learning, a fundamental challenge lies in coordinating heterogeneous features extracted from different encoders. Specifically, audio tokens and video tokens differ not only in modality but also in their temporal structures. To address this, we propose a Frame-level Feature Arrangement module that arranges audio tokens by video-frame index, providing a frame-indexed temporal prior that is convenient for the subsequent causal scan in MFAdapter. By introducing FFA prior to multimodal fusion, the model establishes a more consistent and discriminative representation, which facilitates subsequent cross-modal interaction. We emphasize that FFA is a deterministic, index-based token grouping strategy that assumes the input A-V stream is roughly synchronized, rather than a mechanism that estimates or verifies synchronization. It reduces, but does not eliminate, the coarse segment-level mismatch between audio patches and video frames.

**Audio preprocessing and tokenization.** Following the AudioMAE [45] recipe, each audio stream is resampled to 16 kHz and converted to a log-Mel spectrogram using a 25 ms Hann window with a 10 ms hop, yielding a spectrogram of shape $T_{a}\times F_{a}=1024\times 16$ (time × frequency). The spectrogram is then patchified by AudioMAE with a patch size of 16×16 and a stride of 16×16, producing a spatial grid of (1024/16)×(16/16)=64×1 patches. After the AudioMAE encoder, we therefore obtain one global classification token and $N_{a}^{\mathrm{patch}}=64$ patch tokens, which are flattened row-major (time-major): i.e., token k∈{0,…,63} corresponds to the time interval [10·16k,10·16(k+1))ms. This ordering is fixed and known, so equal partitioning of the flattened sequence is equivalent to equal partitioning along the time axis.

**Frame indexing.** We uniformly sample $N_{v}=16$ frames per clip, and define the temporal center of the *i*-th sampled frame as $t_{i}=\left(i-\frac{1}{2}\right)\cdot \frac{L_{\mathrm{clip}}}{N_{v}}$, where $L_{\mathrm{clip}}\approx 10.24$ s is the clip duration corresponding to the 1024 spectrogram frames. The audio patches are then assigned to frames via nearest-patch assignment: patch *k* (covering time interval [16k,16(k+1)) in spectrogram frames) is assigned to the video frame *i* satisfying $16k\in \left[\left(i-1\right)\cdot \frac{1024}{N_{v}},\;i\cdot \frac{1024}{N_{v}}\right)$. Consequently, each video frame is paired with exactly $N_{a}^{\mathrm{tok}}=N_{a}^{\mathrm{patch}}/N_{v}=64/16=4$ audio patches.

For a video clip of duration $L_{\mathrm{clip}}$, each video frame is associated with a single audio clip of length 1024 spectrogram frames. Given the AudioMAE patch size of 16 spectrogram frames with a 10 ms hop, each audio patch spans 16×10ms=160 ms. With $N_{a}^{\mathrm{tok}}=4$ audio patches assigned per video frame, the total temporal span covered by each frame-aligned audio group is 4×160ms=640 ms. Each sampled video frame represents a temporal interval of $L_{\mathrm{clip}}/N_{v}\approx 640$ ms. The assignment uses interval-boundary matching: audio patch *k* is assigned to video frame *i* if its time interval [16k,16(k+1)) (in spectrogram frames) falls within the *i*-th video frame interval $\left[\left(i-1\right)\cdot 1024/N_{v},\;i\cdot 1024/N_{v}\right)$. This nearest-patch assignment results in a maximum temporal misalignment of ±80 ms, half of a patch width, which is acceptable given the frame-rate granularity. Specifically, the audio patch boundary at 16k (in spectrogram frames) is used for interval-boundary matching with the video frame interval $\left[\left(i-1\right)\cdot 1024/N_{v},\;i\cdot 1024/N_{v}\right]$; the offset between a patch’s center (at 8+16k) and the nearest frame boundary can be at most half a patch width, i.e., ±(8×10ms)=±80 ms. The full audio input (1024 frames ≈ 10.24 s) corresponds to the same clip used for video sampling and is not split into per-frame segments; each frame-aligned audio group merely extracts and reorganizes the relevant patches from this shared audio stream.

**FFA operator.** Given a sequence of audio tokens $F_{a}$ that includes one global classification token and 64 patch tokens, we first split them into:

(10) $$\begin{array}{cc} \; & F_{a}^{cls}=F_{a}\left[:,0:1,:\right]\in R^{B\times 1\times D}, \end{array}$$

(11) $$\begin{array}{cc} \; & F_{a}^{patch}=F_{a}\left[:,1:,:\right]\in R^{B\times 64\times D} \end{array}$$

The patch tokens are equally partitioned into $N_{v}=16$ consecutive temporal segments following the row-major (time-major) ordering above; each segment contains $N_{a}^{\mathrm{tok}}=4$ tokens and corresponds to one sampled video frame:

(12) $$\begin{array}{cc} \; & F_{a}^{PS}=\mathrm{Res}\left(F_{a}^{patch}\right)\in R^{B\times 16\times 4\times D} \end{array}$$

To preserve global semantic information, the same classification token is replicated across frames and concatenated with the patch tokens of each frame ($L_{a}=1+4=5$):

(13) $$\begin{array}{cc} \; & F_{a}^{clsExpand}=\mathrm{Exp}\left(F_{a}^{cls},N_{v}\right)\in R^{B\times 16\times 1\times D}\\ \; & F_{a}^{perFrame}=\mathrm{Concat}\left(F_{a}^{clsExpand},F_{a}^{PS}\right)\in R^{B\times 16\times 5\times D} \end{array}$$

Finally, the batch and temporal dimensions are merged to form frame-level audio representations:

(14) $$\begin{array}{cc} \; & F_{a}=\mathrm{Reshape}\left(F_{a}^{perFrame}\right)\in R^{B\cdot 16\times 5\times D} \end{array}$$

### 3.4. Mamba Fusion Adapter

While FFA provides a frame-level feature arrangement that organizes audio tokens at the video-frame rate, an effective adapter is still required to inject cross-modal cues into the frozen encoders. Traditional fusion approaches often rely on Transformers, where attention captures long-range dependencies but incurs quadratic complexity with sequence length. To overcome this limitation, we design the Mamba Fusion Adapter (MFAdapter) based on the selective state-space model Mamba [43], which offers linear-complexity sequence modeling.

**Figure 2:**
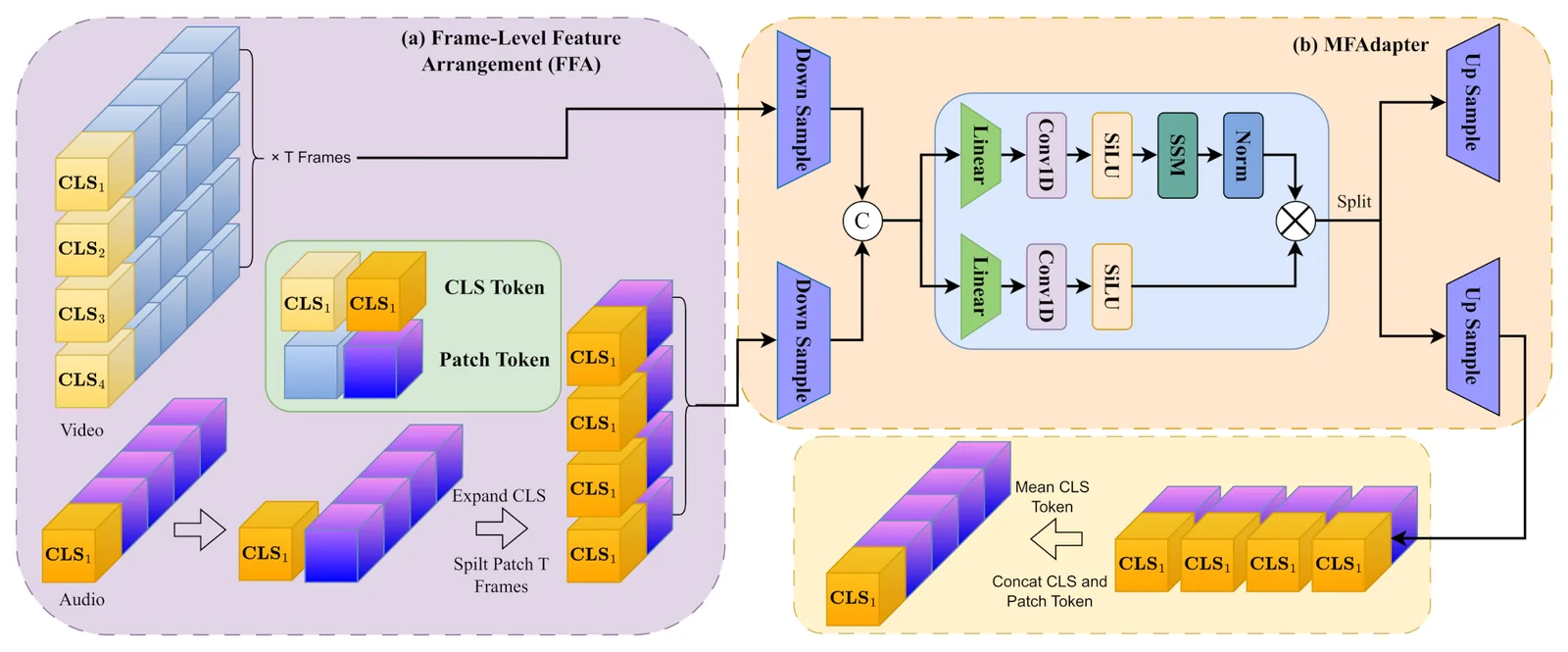
Core module details. (**a**) Frame-level Feature Arrangement (FFA) reorganizes audio tokens to each video frame. (**b**) Mamba Fusion Adapter (MFAdapter): after projection, tokens are concatenated as $\left[F_{a}^{d}\;|\;F_{v}^{d}\right]$ and processed by a causal Mamba scan; outputs are split and projected back to each modality. ${\mathrm{CLS}}_{i}$ denotes the class token of the *i*-th video frame ($i=1,\ldots ,N_{v}$), and CLS denotes the audio class token.

As illustrated in Figure 2, the adapter is an hourglass-shaped module embedded between paired encoder layers. Its pipeline comprises three stages: dimensionality reduction, causal Mamba fusion, and dimensionality restoration.

To place both modalities in a shared latent space, frame-wise audio features $F_{a}$ and video features $F_{v}$ are projected to a reduced dimension $D_{\mathrm{down}}$ by DS(·):

(15) $$\begin{array}{cc} \; & F_{a}^{d},F_{v}^{d}=DS\left(F_{a},F_{v}\right) \end{array}$$

where $F_{a}^{d}\in R^{B\cdot N_{v}\times L_{a}\times D_{\mathrm{down}}}$ and $F_{v}^{d}\in R^{B\cdot N_{v}\times L_{v}\times D_{\mathrm{down}}}$ with $L_{a}\neq L_{v}$ in general. We then form a unified token sequence by concatenating along the token (sequence) dimension, placing audio tokens as a prefix and video tokens as a suffix:

(16) $$\begin{array}{cc} \; & F_{av}^{d}={\mathrm{Concat}}_{\mathrm{seq}}\left(F_{a}^{d},F_{v}^{d}\right)\in R^{B\cdot N_{v}\times \left(L_{a}+L_{v}\right)\times D_{\mathrm{down}}},\\ \; & F_{av}^{\prime }={\mathrm{Mamba}}_{\mathrm{causal}}\left(F_{av}^{d}\right). \end{array}$$

Here, ${\mathrm{Mamba}}_{\mathrm{causal}}$ denotes a standard unidirectional Mamba block with causal convolution and selective scan (left-to-right), rather than a bidirectional or dual-scan variant. Because the hidden state at position *t* depends only on tokens at positions ≤t, this ordering yields an intentionally asymmetric information flow within a single adapter pass: video tokens can condition on the preceding frame-aligned audio context, whereas audio tokens do not observe subsequent video tokens through the scan itself. We adopt this audio-prefix design for DFER because facial dynamics are the primary recognition evidence, while speech prosody provides complementary conditioning that should modulate visual representations when the two streams are temporally aligned by FFA.

After fusion, the output sequence is split back into modality-specific segments of lengths $L_{a}$ and $L_{v}$ along the same token (sequence) dimension that was used for concatenation, taking the first $L_{a}$ tokens as audio and the remaining $L_{v}$ tokens as video:

(17) $$\begin{array}{cc} \; & F_{a}^{\prime },F_{v}^{\prime }={\mathrm{DeConcat}}_{\mathrm{seq}}\left(F_{av}^{\prime };L_{a},L_{v}\right),\\ \; & F_{a}^{\prime }\in R^{B\cdot N_{v}\times L_{a}\times D_{\mathrm{down}}},\;F_{v}^{\prime }\in R^{B\cdot N_{v}\times L_{v}\times D_{\mathrm{down}}}. \end{array}$$

The upsampling operator US(·) restores the dimensionality of each modality:

(18) $$\begin{array}{cc} \; & F_{a}^{\prime \prime },F_{v}^{\prime \prime }=US\left(F_{a}^{\prime },F_{v}^{\prime }\right) \end{array}$$

For the audio branch, $F_{a}^{\prime \prime }$ is first reshaped to $R^{B\times N_{v}\times L_{a}\times D}$; the frame-wise classification tokens are then aggregated across frames by mean pooling:

(19) $$\begin{array}{cc} \; & F_{a}^{cls}=F_{a}^{\prime \prime }\left[:,:,0,:\right]\in R^{B\times N_{v}\times D}\\ \; & F_{a}^{clsMean}=\mathrm{Mean}\left(F_{a}^{cls}\right)\in R^{B\times 1\times D} \end{array}$$

The remaining patch tokens are flattened and concatenated with the aggregated classification token to form the final audio representation:

(20) $$\begin{array}{cc} \; & F_{a}^{patch}=F_{a}^{\prime \prime }\left[:,:,1:,:\right]\in R^{B\times N_{v}\times N_{a}^{\mathrm{tok}}\times D}\\ \; & F_{a}^{patchFlat}=\mathrm{Reshape}\left(F_{a}^{patch}\right)\in R^{B\times N_{a}^{\mathrm{patch}}\times D}\\ \; & F_{a}^{final}=\mathrm{Concat}\left(F_{a}^{clsMean},F_{a}^{patchFlat}\right)\in R^{B\times (1+N_{a}^{\mathrm{patch}})\times D} \end{array}$$

The video branch is directly taken as:

(21) $$\begin{array}{cc} \; & F_{v}^{final}=F_{v}^{\prime \prime }\in R^{B\cdot N_{v}\times L_{v}\times D} \end{array}$$

Finally, residual connections are introduced to preserve pretrained knowledge and stabilize optimization:

(22) $$\begin{array}{cc} \; & F_{a}^{1}=F_{a}+F_{a}^{final},\;F_{v}^{1}=F_{v}+F_{v}^{final} \end{array}$$

Importantly, MFAdapter does not claim symmetric bidirectional interaction inside the causal scan. Instead, hierarchical insertion after every encoder layer progressively strengthens audio-conditioned visual features, while residual write-back to both branches and the subsequent temporal classifier (Section 3.5) provide complementary pathways for integrating multimodal evidence beyond a single unidirectional pass. This design keeps fusion lightweight and explicit about its inductive bias: FFA decides what is temporally comparable, and causal Mamba decides how acoustic context is injected into visual tokens under this alignment.

### 3.5. Classifier Head

The classifier head employs a single-layer Transformer encoder with a hidden dimension of 512 and 8 attention heads. Dynamic facial expression recognition requires on modeling temporal dependencies between consecutive video frames while integrating complementary audio-visual information. In the visual branch, a video sequence containing $N_{v}$ frames is first processed in parallel, and the [CLS] tokens extracted from each frame are concatenated to form a temporal feature sequence. The resulting feature map is reshaped from $B\cdot N_{v}\times L_{v}\times D$ to $B\times N_{v}\times D$, where *B* denotes the batch size, $N_{v}$ the number of frames, $L_{v}$ the token length per frame, and *D* the embedding dimension.

After hierarchical MFAdapter fusion in the encoders, we perform a late temporal aggregation stage that complements the causal audio-prefix scan. The audio [CLS] token is fused with each visual frame token through element-wise addition. The fused representations are then projected through a lightweight linear layer for modality adaptation, reducing computational cost while preserving representational richness. Learnable temporal embeddings and an additional global [CLS] token are incorporated to facilitate temporal reasoning and global context aggregation. The multimodal temporal sequence is subsequently fed into the Transformer layer along the temporal dimension to jointly capture temporal dynamics and cross-modal correlations. The final global representation is obtained from the additional [CLS] token and is forwarded to the emotion classifier.

For optimization, we adopt the standard cross-entropy loss, widely used in multi-class classification. The loss function is defined as:

(23) $$L_{CE}=-\sum_{i=1}^{N}y_{i}\log\left(p_{i}\right)$$

where N denotes the number of emotion classes, y is the ground-truth label, and $p_{i}$ is the predicted probability of the i-th class.

## 4. Experiments

### 4.1. Datasets

(1) DFEW [7]: This dataset is a widely used official in-the-wild benchmark for audio-visual DFER. It comprises 12,059 video clips collected from over 1500 films worldwide, with varying degrees of occlusion, extreme lighting conditions, and pose variations. Each video is annotated ten times by professional annotators and categorized into one of seven basic expressions: happy, sad, neutral, angry, surprised, disgusted, and fearful. The dataset is partitioned into five non-overlapping folds at the clip level, following the official five-fold cross-validation protocol; each fold is used once as the test set, while the remaining four folds are used for training. No temporal sub-segment of any clip is split across folds, so a single video appears in exactly one fold. All DFEW results reported in this paper, follow this fold-level protocol, and the reported numbers are averaged over the five test folds.

(2) MAFW [8]: This dataset is a multimodal database comprising audio-visual and text modalities captured in real-life scenarios. It includes 10,045 audio-visual clips depicting a range of emotional behaviors, each accompanied by a composite emotion category and a brief description of the behavior. Composite emotion annotations include one or more of 11 widely used emotions: anger, disgust, fear, happiness, neutral, sadness, surprise, contempt, anxiety, helplessness, and disappointment. Professional annotators annotated each clip 11 times, and unreliable annotations were filtered out using the Expectation Maximization algorithm. The dataset includes various sources, such as movies, TV shows, news, reality shows, which increases its real-world variability and difficulty. Like DFEW, five-fold cross-validation is used to evaluate the MAFW dataset, with folds defined at the clip level, such that each clip appears in exactly one test fold, and no temporal sub-segment of a clip crosses the train/test boundary. Unless otherwise stated, all MAFW results in this paper follow the official 11-class single-expression classification protocol, consistent with prior multimodal DFER comparisons [15].

### 4.2. Implementation Details

We follow the established protocol of prior works [10,15]: each video is uniformly sampled into 16 frames, and a 2-clip averaging strategy is adopted during inference. The model is trained using AdamW with a base learning rate of $1\times 10^{-3}$, an effective batch size of 16, and a weight decay of $5\times 10^{-2}$. The learning rate follows a cosine decay schedule with 5 warmup epochs, decreasing to a minimum of $1\times 10^{-6}$. A shared FFA module and an MFAdapter are inserted after each paired layer of the video and audio encoders. Specifically, FFA reorganizes audio tokens to match the video frame rate and aligns them with per-frame visual features; MFAdapter then concatenates the aligned audio prefix with video tokens and performs a causal Mamba scan for audio-conditioned cross-modal fusion. The complete hyperparameter configuration is summarized in Table 1. As detailed in Table 2, each MFAdapter adopts a bottleneck dimension of 64, projecting from the 768-d backbone hidden state, a Mamba state dimension of 16, a convolution kernel of 4, and an expand factor of 2, yielding approximately 197.8 K trainable parameters per layer. One MFAdapter is inserted after every Transformer block of both the MAE-Face and AudioMAE encoders (12 layers each), resulting in a total of 12 MFAdapters across the dual-encoder architecture. The parameters of the two frozen unimodal backbones (MAE-Face for video and AudioMAE for audio) remain untouched; only the FFA, MFAdapter, temporal network, and classification head are trainable. As a result, the full model comprises 175.6 M parameters in total, of which only 4.7 M, approximately 2.7%, are updated during training. This PEFT strategy substantially reduces the optimization cost; however, inference still requires loading the two frozen encoders alongside the lightweight fusion modules.

Evaluation is performed using five-fold cross-validation. For each fold, the model is trained on the training split and evaluated on the test split, with the checkpoint achieving the highest weighted average recall (WAR) on the validation set retained for final testing. All experiments are conducted on a single NVIDIA A100 GPU (Nvidia, Santa Clara, CA, USA); each training run completes in approximately 4 h at an input resolution of 224×224. During inference, two clips are uniformly sampled from each video, and their predictions are averaged. Following standard practice in the literature, we report both unweighted average recall (UAR) and weighted average recall (WAR) as evaluation metrics, which are formally defined as follows:

(24) $$\begin{array}{cc} \; & \mathrm{UAR}=\frac{1}{N}\sum_{i=1}^{N}\frac{TP_{i}}{TP_{i}+FN_{i}}\times 100\%\\ \; & \mathrm{WAR}=\frac{\sum_{i=1}^{N}w_{i}\cdot \frac{TP_{i}}{TP_{i}+FN_{i}}}{\sum_{i=1}^{N}w_{i}}\times 100\% \end{array}$$

Here, $TP_{i}$ and $FN_{i}$ represent the number of true positives and false negatives in class *i*, respectively, while $w_{i}$ represents the sample weight associated with class *i*.

**Parameter efficiency vs. computational efficiency.** It is important to emphasize that PEFT reduces the optimization cost—in particular, the gradient buffers and AdamW optimizer-state memory associated with the frozen ViT-Base encoders—but it does *not* eliminate the forward-pass computation through those encoders. The two frozen unimodal backbones still account for the large majority of forward FLOPs and inference latency, because FFA, MFAdapter, and the temporal classifier only add a small fraction of activations on top of the backbone forward pass. As a result, although total and trainable parameters differ by roughly two orders of magnitude in our framework (175.6 M vs. 4.7 M), the gap in forward FLOPs and inference latency is much smaller than the parameter gap; parameter efficiency is therefore not equivalent to computational efficiency.

### 4.3. Comparison with State-of-the-Art Methods

We compare our proposed method with existing audio-visual DFER methods on the DFEW [7] and MAFW [8] datasets. We explicitly acknowledge that MMA-DFER achieves higher absolute WAR/UAR (77.51%/67.01%) on DFEW than our method (76.62%/65.25%). On MAFW, our method attains the best WAR (58.70%) and the second-best UAR (43.66%) among the compared methods; the WAR margin over MMA-DFER (58.52%) is only 0.18 pp, while our UAR (43.66%) trails MMA-DFER’s UAR (44.11%) by 0.45 pp. As detailed in Table 3, this 0.18 pp WAR margin is comparable to the cross-fold standard deviation on MAFW, so we do not claim statistically significant superiority over MMA-DFER on MAFW. We therefore position our method as a competitive PEFT-based audio-visual DFER approach that achieves better performance than the full fine-tuning baselines (HiCMAE: 75.01%/63.76% on DFEW; 56.17% WAR on MAFW), while using only 4.7 M trainable parameters (2.7% of total).

**DFEW.** Table 4 compares the proposed method with state-of-the-art (SOTA) methods on the DFEW dataset. Following the standard protocol of previous work, we adopt the official five-fold cross-validation scheme and repeat each fold with three independent random seeds, yielding a total of 15 runs (5folds×3seeds). Throughout this paper, the primary reported performance is the cross-fold mean computed over the five official test folds (DFEW: 76.62% WAR/65.25% UAR; MAFW: 58.70% WAR/43.66% UAR). To provide uncertainty estimates, we additionally run each fold with three random seeds and report the overall mean ± standard deviation over all n=15 runs in Table 3. The two sets of values are consistent: the cross-fold mean matches the overall 15-run mean to within 0.29 pp for all four metrics, confirming experimental stability. To avoid ambiguity between model capacity and optimization cost, we separately report the total parameters and the trainable parameters: our method loads 175.6 M parameters at inference but updates only 4.7 M during training. Across the 15 runs, our method attains a WAR of 76.55%±1.20%, a UAR of 65.12%±1.23%, and a weighted F1 of 76.71%±1.57% (mean ± std, n=15); the 95% CIs of the mean are [75.89%,77.21%] for WAR and [64.44%,65.80%] for UAR. As shown in Table 3, folds 1–5 are individually stable (per-fold std ≤ 0.81 pp). We explicitly acknowledge that MMA-DFER achieves 77.51%/67.01% (WAR/UAR), exceeding our method by 0.96 pp in WAR and 1.76 pp in UAR. Nonetheless, our method updates only 4.7 M trainable parameters, 37% fewer than MMA-DFER’s 7.5 M, while achieving better WAR than full fine-tuning baselines (HiCMAE: 75.01%/63.76%).

**MAFW**. As detailed in Section 4.1, MAFW provides 11 emotion categories. Table 4 reports results under the official 11-class single-expression setting with five-fold cross-validation. The clips exhibit rich variations in lighting, posture, and occlusion, reflecting practical in-the-wild challenges.

To match the variability analysis provided for DFEW and to address the reviewer’s request for uncertainty estimates on MAFW, we report per-fold WAR/UAR/WF1, together with the cross-fold mean, standard deviation, and 95% confidence interval of the mean over the five official MAFW folds, in Table 3 (bottom rows), using the same 5-fold × 3-seeds protocol as that used for DFEW (n=15 in total). Across the 15 runs, our method attains a WAR of 58.41%±5.71% and a UAR of 43.61%±5.10% (mean ± std, n=15); the 95% CI of the mean is [55.26%,61.57%] for WAR and [40.79%,46.44%] for UAR. Per-fold std on MAFW ranges from 0.08 to 1.19 pp, and the WAR advantage over MMA-DFER (0.18 pp) is well below the cross-fold standard deviation (5.71 pp); we therefore do not claim statistically significant superiority over MMA-DFER on MAFW WAR. Our method achieves the highest WAR (58.70%) and the second-highest UAR (43.66%) among all compared methods in Table 4 (per-fold statistics and 95% CIs are reported in Table 3). In particular, given that the WAR advantage over MMA-DFER is comparable to the typical cross-fold variation, we refrain from claiming statistically significant superiority on MAFW WAR; instead, we position our method as competitive among PEFT-based audio-visual DFER methods under the official five-fold cross-validation protocol.

Each MAFW fold is run with the same 5-fold × 3-seeds protocol as DFEW (n=15 in total), reusing the available seeds and reporting the cross-fold mean ± std together with the 95% CI of the mean in Table 3; the 95% CI of the mean is wider for MAFW than for DFEW, ±3.16 pp vs. ±0.66 pp for WAR, because MAFW exhibits greater fold-level variability under the official five-fold protocol.

As shown in Table 5, the model without oversampling achieves a clear improvement in UAR by 1.84%, while the WAR increases only slightly by 0.64%. This discrepancy mainly arises from the uneven performance of the model across different emotion categories. UAR, which averages recall across all classes, is more sensitive to minority emotions, such as fear and disgust, whereas WAR is dominated by majority categories with larger sample sizes, such as neutral and happy.

On the DFEW dataset, the proposed MFAdapter and FFA effectively enhance the model’s representational capacity and discriminative ability, particularly improving recognition performance for underrepresented emotion categories. This leads to a more balanced recall distribution across classes and a clear rise in UAR. However, since majority categories already exhibit strong recognition accuracy, their large sample weights contribute less to the overall WAR improvement. These results indicate that the proposed method achieves more balanced emotion recognition on the DFEW dataset under the official seven-class protocol, rather than merely improving overall accuracy, yielding a more even recall distribution across the official category set.

To provide a finer-grained view of class-wise behavior on DFEW, we further report per-class results in Table 5, complementing the aggregate UAR/WAR comparison in Table 4.

### 4.4. Ablation Studies

To thoroughly validate the effectiveness of the proposed method, we conducted a comprehensive set of ablation studies on the DFEW dataset. Starting from an MAE-Face [46] and AudioMAE [45] baseline, we systematically evaluated different MFAdapter configurations and compared various multimodal fusion strategies. The following experiments analyze the contribution of each component in detail.

**Adapter effectiveness across modalities.** To evaluate the effectiveness of unimodal (video-only and audio-only) versus multimodal (audio-visual) inputs, we conducted experiments under two training configurations: (1) freezing the backbone network parameters and updating only the classifier, and (2) freezing the backbone parameters while updating both the adapter and the classifier. As shown in Table 6, the video modality consistently outperformed the audio modality across all experiments, indicating that visual cues provide stronger discriminative power for DFER than audio cues. Furthermore, multimodal input surpassed unimodal input in performance, confirming the complementary nature of visual and auditory information in capturing fine-grained emotional representations. Notably, MFAdapter achieved clear performance improvements in both unimodal and multimodal settings, validating its effectiveness.

**Audio robustness under controlled interventions.** To isolate the contribution of the audio stream, we ran controlled ablations on DFEW that kept video frames, labels, model capacity, optimizer, and training schedule unchanged: muted replaces each aligned waveform with silence before fbank extraction, while permuted randomly shuffles four contiguous segments using a sample-wise reproducible key. We additionally introduce a capacity_control condition, in which MFAdapter is replaced by a constant-token branch that has the same trainable parameter count, train_muted in which audio is replaced by silence only during training, and test_time_mute in which a normal-audio checkpoint is evaluated with silence substituted for audio at inference time only. As shown in Table 7, three observations follow.

(i) Capacity-matched control. The capacity_control branch, a constant-token adapter with 4.7 M trainable parameters, yields 59.71%/73.17% UAR/WAR, only 1.81/2.00 pp above the video-only adapter baseline (57.90%/71.17%). The full muted condition, evaluated under the same parameter budget, reaches 62.42%/75.65%. The 2.71/2.48 pp additional gain over capacity_control therefore cannot be attributed to additional trainable parameters, constant features acting as learned biases, or to the architectural asymmetry between the video-only and audio-visual branches: the only difference between capacity_control and muted is the content of the audio tokens. We describe the muted-audio result as evidence that the frozen AudioMAE encoder, after per-channel normalization, produces a non-trivial representation on silent input that is distinguishable from a constant-token control and that interacts differently with the trained fusion architecture. We do not, however, interpret this as direct evidence that the silent input itself carries usable acoustic or class-specific information: the experiment isolates the role of the audio-token content, but does not by itself characterize what (if any) class-related structure the silent encoding contains.

(ii) Training-time vs. test-time muting. train_muted (59.19% UAR) almost matches the video-only adapter baseline (57.90% UAR), suggesting that a model that has never been exposed to audio cannot learn to rely on audio features and effectively collapses to a video-only classifier. By contrast, test_time_mute (63.54% UAR) is only 1.71 pp below the full model (65.25% UAR), and still 1.83 pp above the capacity_control branch. These two experiments answer different questions and should not be conflated: train_muted tests whether the audio stream is necessary during learning, while test_time_mute tests whether a fully trained audio-visual model retains usable audio-conditioned features once the acoustic content is removed at inference.

(iii) Temporal ordering. The difference between permuted and muted is only 0.55 pp UAR, while the difference between the full model and permuted is 2.28 pp UAR. We describe this result cautiously: temporal ordering of audio features is a secondary contributor on top of the audio stream, and frame-level alignment is therefore a refinement rather than a primary driver of the audio-visual gain.

**Figure 3:**
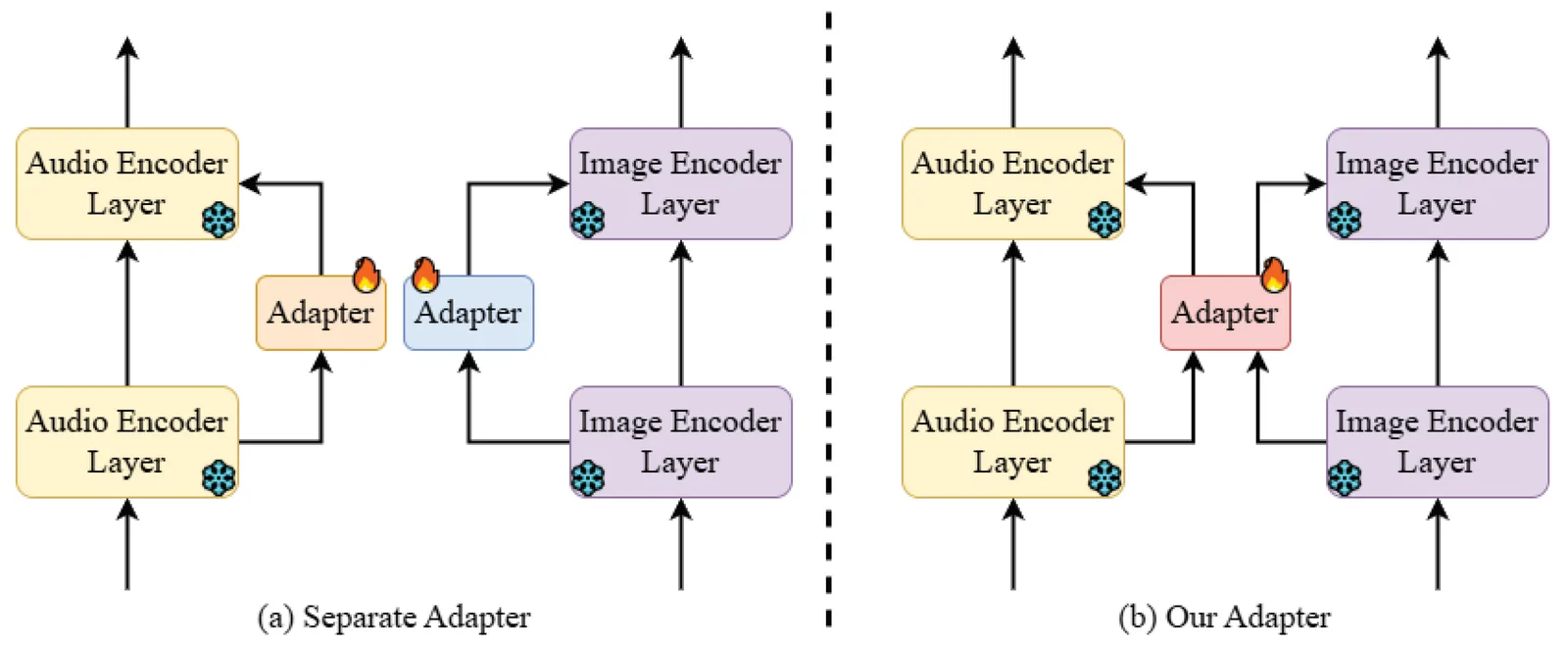
Architecture comparison: Separate Adapters vs. MFAdapter.

**MFAdapter versus separate adapters.** To validate the design advantages of MFAdapter, we implement a separate-adapter baseline within the same pretrained backbone framework. Unlike the unified MFAdapter, which fuses multimodal features at an intermediate stage, this baseline deploys two separate modality-specific adapters (one for video, one for audio), as depicted in Figure 3. Consistent with previous experiments, the backbone parameters remain frozen, and only the adapter modules and the classification head are updated. As shown in the quantitative comparison presented in Table 8, MFAdapter demonstrates clear superiority over this independent-adapter approach on key metrics (e.g., +5.57% UAR, +3.40% WAR). These results indicate that joint causal fusion over the concatenated audio-visual sequence is more effective than updating two modality-specific adapters independently and deferring interaction to a later stage.

**Effect of Adapter Number and Position.** To identify efficient MFAdapter configurations, we systematically evaluate variants inserted after different subsets of encoder layers within the backbone. The experimental results in Table 9 show consistent performance gains across all placements, confirming the architectural robustness of our design. Notably, placing adapters after every encoder layer yields the best classification performance, which we attribute to deeper multilevel feature integration across Transformer stages. This suggests that progressive audio-conditioned adaptation across all encoding depths most effectively enhances task-relevant visual representations.

**Effect of Frame-level Feature Arrangement.** We systematically evaluate feature fusion strategies within the main framework to identify an efficient scheme for feature reorganization. As shown in Table 10, our method outperforms simple concatenation; the FFA grouping strategy provides a frame-indexed token-grouping prior that the subsequent MFAdapter scan can consume directly, rather than claiming strictly verified audio–visual alignment. This design enables feature interaction in a temporally structured space instead of through unstructured concatenation, leading to stronger task-relevant representations across adapter layers.

**Effect of Adapter Variants on Performance.** The MFAdapter is the sole learnable bridge between the frozen audio and visual encoders, making its fusion operator the most critical inductive-bias choice. We therefore replace its Mamba selective scan with three learnable alternatives plus an Identity baseline—an MLP bottleneck, a cross-attention encoder, a LoRA-style low-rank adapter, and an Identity mapping (no fusion)—under a matched PEFT budget of 4.2–4.7 M trainable adapter parameters (frozen backbone fixed at 175.6 M). Two observations follow from Table 11. Cross-modal interaction is necessary: the Identity mapping (UAR 60.76, WAR 72.96) trails the best MFAdapter (UAR 65.25, WAR 76.62) by 4.49/3.66 percentage points, while all learnable operators remain above Identity. Among learnable operators, the causal Mamba scan performs best, exceeding the MLP bottleneck by 1.90/1.70 percentage points, the cross-attention encoder by 4.19/2.55 percentage points, and the LoRA adapter by 1.32/1.61 percentage points. The MLP and LoRA variants cluster tightly (UAR 63.35 vs. 63.93, WAR 74.92 vs. 75.01): both apply token-wise channel mixing without sequence modeling, and the small gap reflects operator structure rather than trainable budget (MLP 4.3 M, LoRA 4.2 M). On the FFA-aligned token sequence, bidirectional cross-attention is the weakest learnable operator (UAR 61.06, WAR 74.07), underperforming the parallel MLP by 2.29/0.85 percentage points despite carrying the largest adapter budget (4.6 M). This indicates that content-dependent, causally ordered state transitions along the FFA scan direction—exactly what the Mamba selective scan provides—match the FFA-imposed temporal structure better than bidirectional query–key mixing. Taken together, a causally ordered, content-adaptive state-space scan is the most appropriate fusion operator for our FFA-aligned framework.

**Effect of Down-sampling Dimensions $D_{down}$.** We investigate the impact of the down-sample dimension on model performance. This parameter determines the feature dimensionality before and after the MFAdapter fusion, thereby directly influencing both representational capacity and computational cost. To analyze the role of $D_{down}$ in classification performance, we conducted comparative experiments under identical training settings, as summarized in Table 12.

The results show that increasing $D_{\mathrm{down}}$ from 32 to 64 leads to a notable improvement in accuracy, indicating that overly small feature spaces cause information loss and insufficient representation. However, as the dimension continues to increase, the number of trainable parameters increases approximately linearly with the adapter width (and more steeply for wider bottlenecks due to the projection matrices), while accuracy gains become marginal, suggesting that the model’s representational capacity has reached saturation. Moreover, larger dimensions incur higher computational costs, and a moderate dimension (around $D_{\mathrm{down}}=64$) achieves the best trade-off between performance and efficiency, preserving sufficient multimodal information while maintaining lightweight computation.

**Efficiency Gains Beyond Parameter Count.** Crucially, the Mamba-based linear complexity of MFAdapter is a property of the adapter-level fusion pathway, not of the overall architecture: the frozen MAE-Face and AudioMAE backbones remain ViT-Base Transformers whose quadratic self-attention still dominates the end-to-end forward cost. Besides the 97.3% reduction in trainable parameters, the PEFT paradigm also yields substantial GPU memory savings. As shown in Table 13, the peak training memory under the same batch size of 8 drops from 22.1 GB (full fine-tuning) to 14.1 GB (MFAdapter), a 36.2% reduction. The saving originates from the absence of gradients and AdamW optimizer states for the 171.0 M frozen backbone parameters, as well as the reduced activation memory for the frozen encoder blocks. Inference latency (55.24 vs. 55.28 ms per sample) is unchanged, because all 175.6 M parameters must still be loaded and computed at test time regardless of which subset was updated during training. Inference peak memory drops from 2.42 GB (full fine-tuning) to 1.76 GB (MFAdapter), a 27% reduction; the saving is *not* due to loading a smaller subset of weights (the same 175.6 M parameters are loaded in both configurations). Because inference runs under model.eval() with torch.no_grad(), the autograd graph is not constructed at all, so (i) the param.requires_grad flag does not influence activation caching, and (ii) gradient checkpointing—a training-time technique that recomputes activations during the backward pass—is not applicable during inference. The remaining 0.66 GB gap should therefore be interpreted as a residual allocator-level difference attributable to allocator state, cuDNN/attention workspace variance, and minor run-to-run numerical differences under matched architecture, precision and inference mode, rather than as evidence of a specific PEFT-related inference-time memory-saving mechanism. In practice, the inference footprints of PEFT and full fine-tuning are comparable. Both runs use FP32 precision and identical forward architectures (see the caption of Table 13 for the full measurement protocol). These results confirm that the PEFT advantage is primarily a training-time efficiency benefit: at batch size 8, the 14.1 GB MFAdapter footprint leaves a much larger headroom on the A100 for higher batch sizes or longer clips than the 22.1 GB full fine-tuning counterpart.

**Effect of Full Fine-Tuning Under Matched Training Settings.** To isolate the contribution of the interaction modules from the effect of backbone adaptation, we further compared our PEFT strategy with a full fine-tuning scheme that keeps every other training configuration identical (optimizer, schedule, batch size, and number of epochs), in which both the multimodal backbones and the adapter modules are updated simultaneously. Importantly, the peak learning rate is re-tuned within each paradigm to its empirical optimum, because PEFT updates only auxiliary modules whereas full fine-tuning modifies the entire backbone and would otherwise suffer from catastrophic forgetting at aggressive rates. As reported in Table 14, the two configurations share the same total capacity at inference (175.6 M), yet only 4.7 M parameters are trainable in our PEFT setting, a reduction of approximately 97.3% in the trainable-parameter budget. Under this drastically reduced trainable budget, our PEFT setting still achieves a higher UAR (**65.25** vs. 63.39, Δ=+1.86) at the cost of a slightly lower WAR (76.62 vs. **77.53**, Δ=−0.91).

The opposite trends on UAR and WAR are precisely the diagnostic signal we expect from a parameter-efficient design: WAR, dominated by majority classes such as Happy and Neutral, marginally favors full fine-tuning, which retains sufficient capacity to over-fit the most frequent categories on a small annotated set such as DFEW; UAR, which weights every class equally, instead rewards the balanced recall produced by our frozen-backbone design. Because the learning rate was independently re-tuned to its empirical optimum within each paradigm, the residual gaps of ΔUAR and ΔWAR in Table 14 can be attributed to the parameter-count gap itself rather than to any training-intensity mismatch. Three factors account for this behavior. *Preservation of pretrained representations.* Freezing the MAE-Face and AudioMAE encoders keeps the large-scale self-supervised knowledge intact and prevents the backbone from drifting toward dataset-specific shortcuts, which is particularly harmful for minority emotions. *Concentration of optimization on the fusion path.* With only the FFA, MFAdapter, and temporal network trainable, gradients are forced to flow exclusively through the cross-modal interaction pathway, so the learning capacity is invested where the task actually demands it rather than being diluted across 175.6 M parameters. *(Implicit regularization by low parameter count.)* The 4.7 M trainable budget acts as a strong inductive bias that discourages majority-class dominance in the decision boundary, thereby improving recall on rare categories such as Disgust and Fear. Consequently, the WAR gap of −0.91 is a small and acceptable trade-off given the 37-fold reduction in trainable parameters and the much larger +1.86 UAR improvement, which directly translates into more equitable recognition across all emotion categories—as also reflected in the t-SNE and confusion-matrix analyses. These findings reinforce that, for in-the-wild DFER, performance gains are driven primarily by effective cross-modal interaction modeling rather than by exhaustively updating the unimodal backbones, and that parameter-efficient tuning is not merely a computational convenience but a principled strategy for balanced multimodal recognition.

### 4.5. Visualization Analysis

**Figure 4:**
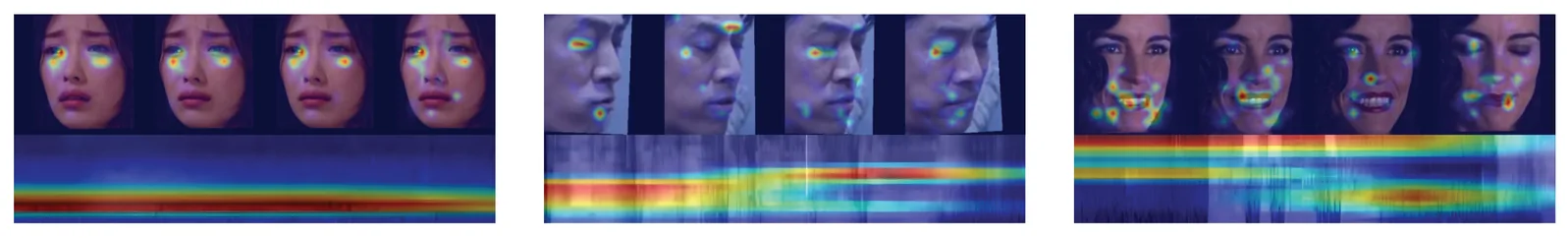
Attention visualization of the proposed method on the DFEW dataset for Sad, Neutral, and Happy emotion categories. The first row illustrates the visual-domain Grad-CAM responses on input video frames, while the second row presents the audio-domain attention distributions over the Log-Mel spectrogram.

**Attention Visualization.** To further validate the effectiveness of the proposed method, we visualize the attention distributions of the final Transformer module, as shown in Figure 4. The figure comprises two components: Grad-CAM heatmaps for the visual modality and attention maps over the log-Mel spectrogram for the audio modality. The log-Mel representation applies logarithmic compression to Mel-scaled energy, producing smoother amplitude variations that align with the nonlinear loudness perception of the human auditory system. This facilitates the observation of rhythmic changes, pitch contours, and energy patterns, making it suitable for capturing fine-grained acoustic cues in emotion recognition. Based on this representation, three representative emotion categories are selected to compare the attention distributions across audio and visual modalities and to reveal the model’s cross-modal feature extraction behavior.

The visualizations reveal clear differences in attention patterns and cross-modal responses across the Sad, Neutral, and Happy samples. For the Sad sample, visual attention concentrates around the eyes and glabellar region, indicating that the model effectively captures muscle activations associated with sadness. The audio attention in the low-frequency regions is consistent with the typical spectral characteristics of sad speech. In the Neutral sample, visual attention becomes more compact around the eyelids and eyebrows, with stronger activation during eye closure, reflecting the model’s sensitivity to subtle facial variations. The audio modality exhibits stable low-frequency responses, while slight mid-frequency activations occur synchronously with minor facial movements, demonstrating cross-modal consistency during weak expression transitions. For the Happy sample, visual attention prominently covers the mouth corners, cheeks, and periocular regions, expanding temporally as the smile intensifies. Meanwhile, the audio attention in the mid- and high-frequency regions is consistent with the typical spectral characteristics of positive speech. Furthermore, as the smile recedes and facial movements change, the audio attention patterns on the spectrogram exhibit corresponding temporal variations, highlighting strong dynamic consistency between the visual and audio modalities.

Overall, the multimodal visualization results demonstrate that the model reliably identifies emotion-related regions in both modalities and maintains coherent temporal correspondence across them. This confirms the effectiveness of the proposed framework in cross-modal dynamic modeling and emotional semantic fusion.

**Figure 5:**
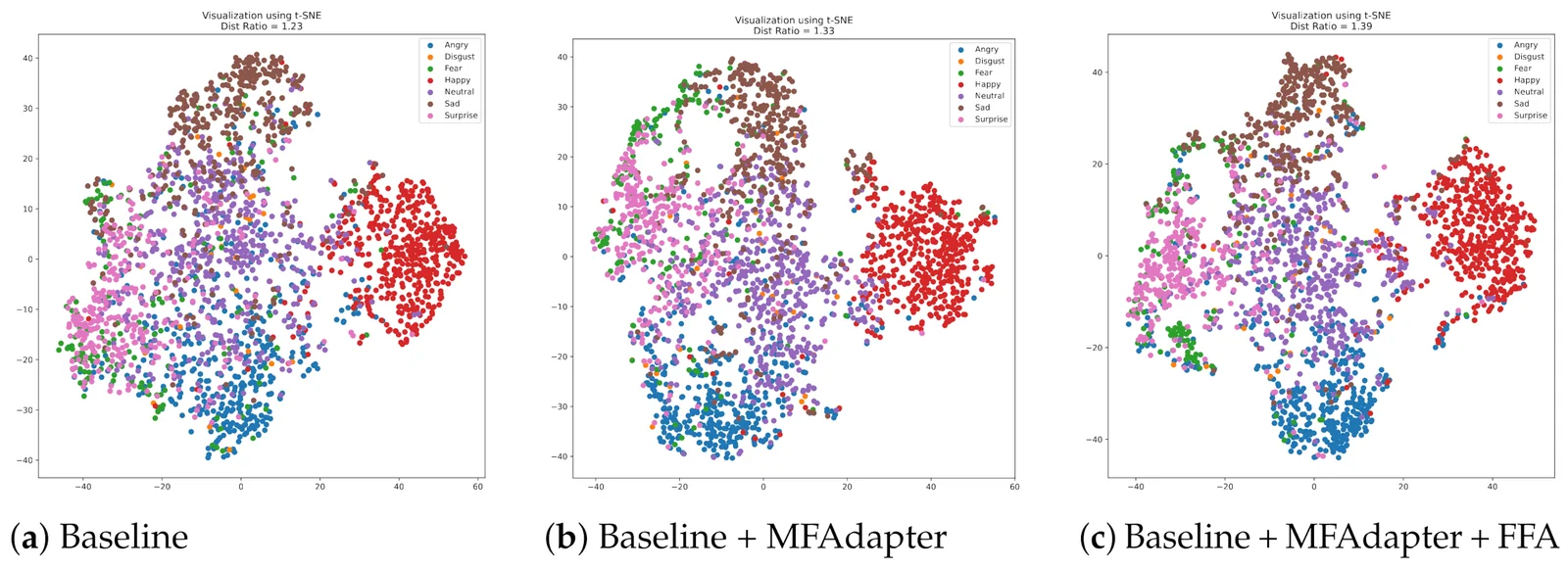
t-SNE visualization on DFEW for (**a**) baseline, (**b**) +MFAdapter, (**c**) +MFAdapter + FFA. Hyper-parameters): perplexity =40, lr =200, $n_{\mathrm{iter}}=1000$, full val. set, PCA init, seed 42, raw features (no L2 norm). Overlaid numbers are 2-D trend references only, not rigorous metrics.

**Visualization of Feature Distribution.** As shown in Figure 5, we progressively incorporate our proposed components into the baseline model and visualize the high-level features on the DFEW test set using t-SNE. From (a) to (c), the per-class clusters become visually more compact, and the overlap between adjacent emotions (e.g., Neutral vs. Sad) gradually decreases, which is consistent with the quantitative trends reported in Table 6 and Table 10. We caution that any geometric reading of the 2-D embedding should be regarded as an illustrative trend, not as a rigorous separation metric, because t-SNE does not preserve global distances. Notably, the Happy class exhibits clear boundaries, highlighting the ability of our components to learn discriminative features. Although overlaps remain for minority classes (e.g., Disgust) due to data imbalance, the results further emphasize the robustness of our approach in capturing fine-grained emotional representations through multimodal fusion.

**Figure 6:**
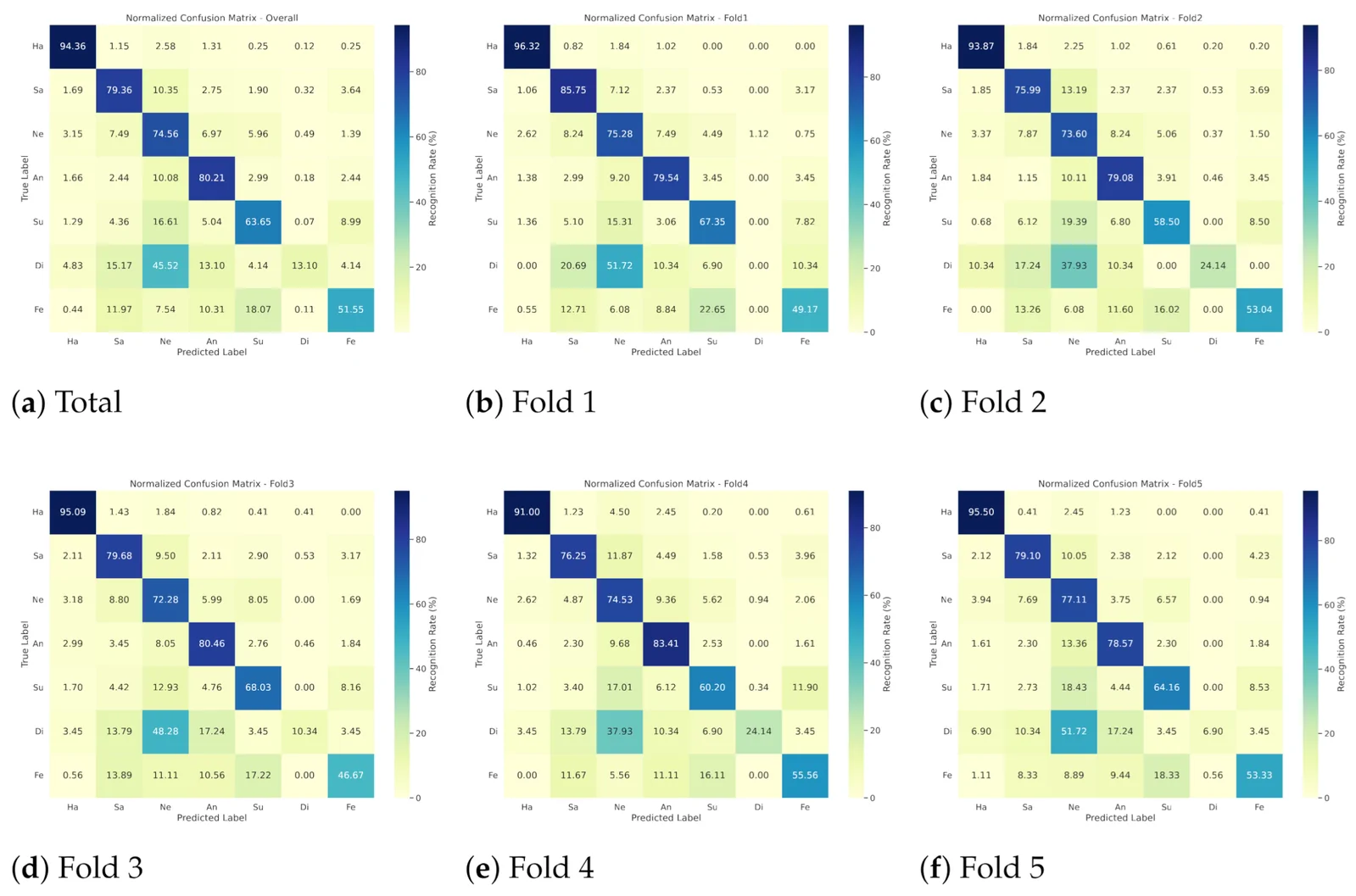
Confusion matrices of the DFEW dataset across different folds. (**a**) shows the average confusion matrix, while (**b**–**f**) show results for individual folds.

**Visualization of Confusion Matrix.** As illustrated in Figure 6, the proposed method demonstrates stable and consistent performance on the DFEW dataset. The averaged confusion matrix in (a) shows that the model performs strongly on high-intensity emotion categories such as happy, angry, and sad, all achieving recognition rates above 75%. In particular, the happiness category reaches about 94%, indicating the model’s strong capability in capturing salient emotional patterns. Meanwhile, for semantically similar or fine-grained emotions such as neutral and disgust, which are inherently harder to distinguish due to subtle feature differences and data imbalance, the model still learns meaningful discriminative representations, reflecting its robustness in handling complex emotional nuances. Further examination of the five-fold cross-validation results in (b)–(f) reveals that the remaining six emotion categories are stable across folds, demonstrating that the model’s generalization behavior is consistent for the majority of the label set. For the Disgust category, however, the fold-level recognition is markedly less stable: Fold 1 yields approximately 0%, while Folds 2 and 4 reach approximately 24%. This large cross-fold variation is consistent with Disgust being the rarest class in DFEW and therefore the most susceptible to fold-level sampling; it does not contradict, but rather instantiates, the class-imbalance limitation stated below, and we therefore restrict our cross-fold stability claims to the six non-minority emotions. Overall, the proposed approach effectively distinguishes prominent emotions while also showing promising results for subtle emotional categories, underscoring its robustness and adaptability. These findings suggest that further extending this framework, such as by incorporating additional modalities or exploring hierarchical emotion structures, could enhance its applicability in more complex affective computing scenarios.

**Figure 7:**
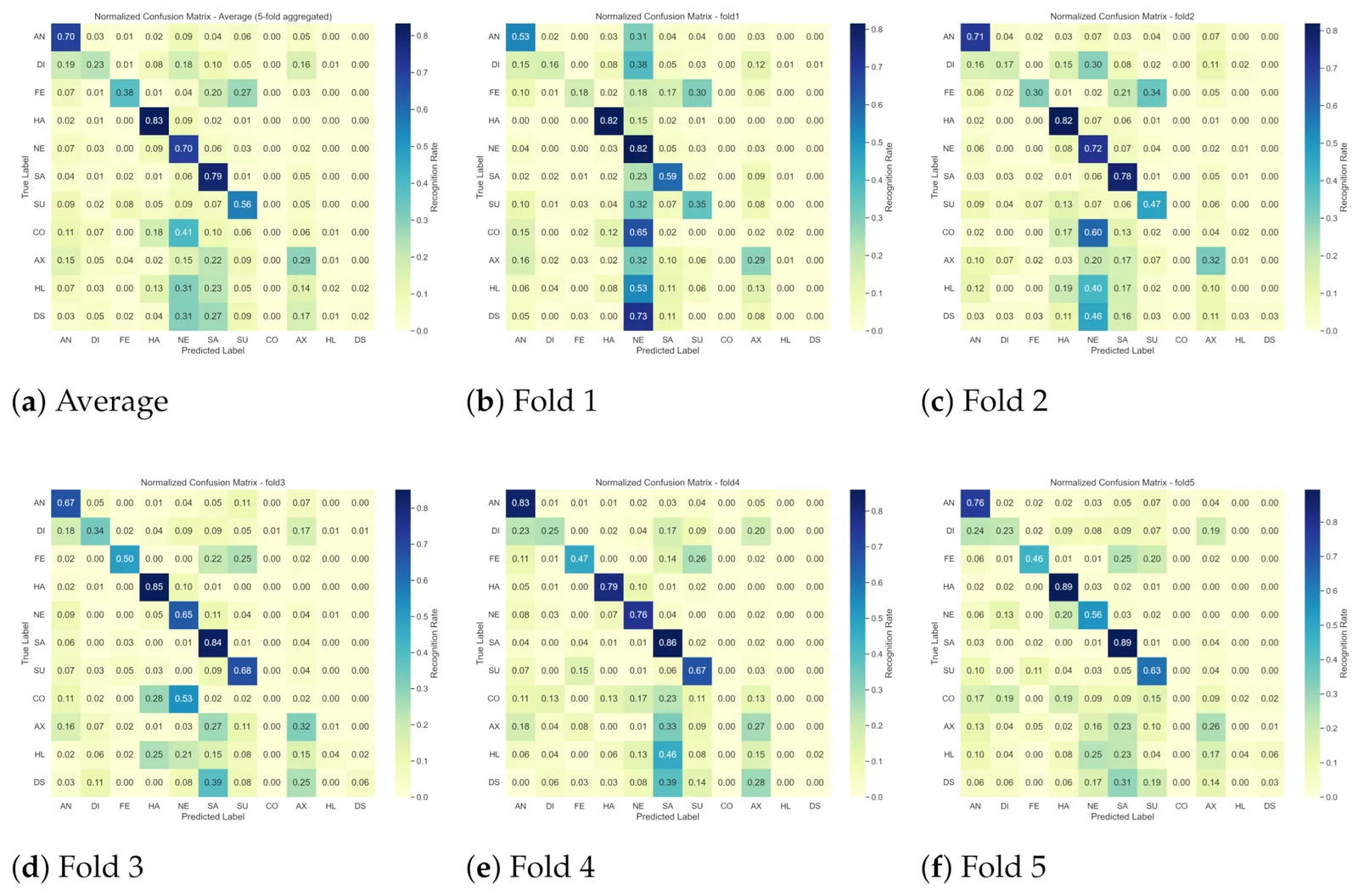
Confusion matrices of the MAFW dataset across different folds. (**a**) shows the average confusion matrix obtained by aggregating the five folds (counts summed across folds and then row-normalized), while (**b**–**f**) show results for individual folds.

For completeness, we further analyze the per-fold and averaged confusion matrices on the MAFW dataset (Figure 7), which has an 11-class taxonomy with a substantially longer tail than DFEW. The aggregated matrix in (a) and the five-fold breakdown in (b)–(f) reveal three characteristic patterns. **First**, salient and high-intensity emotions remain reliably recognized across all folds: Happy achieves 81.5–89.1% (mean 83.4%) and Sad rises monotonically from 58.8% in Fold 1 to 89.5% in Fold 5 (mean 79.4%), confirming that the audio–visual cues learned on pretrained models transfer cleanly to MAFW. **Second**, medium-frequency emotions show a consistent cross-fold improvement: Surprise climbs from 34.7% (Fold 1) to 67.8% (Fold 3) and stabilizes at 63–67% in Folds 3–5, indicating that the framework progressively disambiguates Surprise from visually similar Neutral and Happy expressions; Fear likewise improves from 17.6% (Fold 1) to 49.6% (Fold 3) and stays above 45% afterwards. **Third**, the long-tail emotions remain hard—Contempt, Helpless and Disgust each have per-fold recall below 5%—which is consistent with their severely under-represented sample counts (typically fewer than 50 clips per fold) and their strong visual and acoustic overlap with Neutral, rather than with the proposed method. Notably, Angry also fluctuates across folds (55.0–83.1%), reflecting its well-known confusion with Sad in the wild. Overall, the MAFW matrices show that the discriminative behavior is stable across folds for major emotions and improves monotonically for medium-frequency ones, while residual errors concentrate on the expected long-tail classes—the same balanced-recall pattern observed on DFEW, but more pronounced due to MAFW’s finer label set.

## 5. Limitations and Responsible Use

This study has four main limitations. First, DFEW and MAFW are drawn from films and television programs, so their distributions may not represent all ages, ethnicities, genders, cultures, or languages. Aggregate UAR and WAR therefore do not guarantee equitable performance across demographic subgroups, and subgroup-specific analysis is needed before any deployment.

Second, class imbalance and subtle inter-class boundaries, e.g., neutral versus disgust, persist: the confusion matrices show that minority or fine-grained emotions remain harder to recognize, which constrains recognition reliability in these cases.

Third, the framework assumes synchronized audio-video streams. FFA employs deterministic index-based arrangement and is sensitive to: variable clip durations, non-uniform frame rates, missing/silent audio, boundary truncation, and A-V temporal offsets. In practice, DFEW/MAFW clips are pre-aligned at the dataset level. FFA is a deterministic index-based arrangement that provides a frame-indexed temporal prior, not a verified frame-level synchronization mechanism. Future work will explore soft alignment and cross-dataset transfer. Ablation scope. All ablations are conducted on DFEW under five-fold cross-validation to validate general architectural choices; consistent gains across folds, together with strong MAFW results, jointly support the effectiveness of our design.

Fourth, the scope of our SOTA claims is intentionally narrow. On DFEW, our method is not the top-performing approach: MMA-DFER exceeds our WAR/UAR by 0.96/1.76 pp while updating 7.5 M trainable parameters; on MAFW, our method achieves the best WAR (58.70%) and the second-best UAR (43.66%) among compared methods, exceeding MMA-DFER’s WAR (58.52%) by only 0.18 pp while trailing it in UAR by 0.45 pp. Given that this gap is comparable to the typical cross-fold standard deviation on MAFW (Table 3), we do not claim statistically significant superiority over MMA-DFER on MAFW. Consequently, the abstract and conclusion describe the method as achieving competitive performance on DFEW and MAFW while updating 2.7% of the model parameters, rather than as state-of-the-art on multiple benchmarks. To enable readers to judge the practical uncertainty, we report the per-fold mean ± std and 95% CI of the mean for both datasets; MAFW follows the same 5-fold × 3-seed protocol as DFEW, yielding n=15 runs, whose per-fold and overall statistics are reported in Table 3.

## 6. Conclusions

In this work, we present a parameter-efficient audio-visual framework for in-the-wild DFER. By freezing encoder parameters and training lightweight interaction modules, the proposed framework preserves pretrained unimodal representations and concentrates optimization on the cross-modal fusion pathway. To this end, we introduce Frame-level Feature Arrangement (FFA), a deterministic index-based arrangement strategy that arranges audio tokens at the video-frame rate. FFA does not by itself verify frame-level stream synchronization; instead, it provides a frame-indexed temporal prior that supports the ordered causal scan inside MFAdapter and reduces, but does not eliminate, coarse segment-level mismatch. Building on this frame-indexed grouping, MFAdapter performs causal selective scanning over an audio-prefixed token sequence, enabling efficient audio-conditioned visual adaptation at multiple encoder depths with only a small number of trainable parameters. Complementary late temporal fusion further aggregates multimodal evidence for recognition. Extensive experiments on the DFEW and MAFW benchmarks demonstrate that the framework achieves competitive performance while updating 2.7% (4.7 million) of the model parameters. Specifically, our method attains 76.62% WAR/65.25% UAR on DFEW, with the mean ± standard deviation over n=15 runs reported in Table 3, and 58.70% WAR/43.66% UAR on MAFW, with the cross-fold mean over the five official folds reported in Table 3. We position our method as a competitive PEFT-based audio-visual DFER framework rather than as state-of-the-art on multiple benchmarks: on DFEW, it is surpassed by MMA-DFER in absolute WAR/UAR while updating 37% fewer trainable parameters, and on MAFW, it exceeds MMA-DFER’s WAR by only 0.18 pp while trailing its UAR by 0.45 pp—a margin comparable to the cross-fold standard deviation on MAFW; therefore, we do not claim statistically significant superiority. Per-fold mean ± std and the 95% CI of the mean for both datasets are reported in Table 3 to enable readers to judge the practical uncertainty of the comparison. We note that the current work is evaluated strictly within benchmark-based expression recognition settings; extension to sensitive domains such as healthcare or clinical support remains a promising future direction, pending rigorous domain-specific validation and ethical safeguards.

## Figures and Tables

**Table 1:** Key experimental settings for the proposed audio-visual DFER framework.

Data Processing	Value	Training Configuration	Value
Frames per video	16	Batch size	16
Sampling rate	4	Learning rate	$1\times 10^{-3}$
FFT hop length (ms)	10	Epochs	25
Mel spectrogram bins	16	Optimizer	AdamW
Input resolution	224 × 224	Min LR	$1\times 10^{-6}$
Audio sampling rate (Hz)	16,000	Weight decay	$5\times 10^{-2}$
Audio input length (frames)	1024	Warmup epochs	5
FFT window size (ms)	25	Beta_1_, Beta_2_	0.9, 0.999
Model Architecture	Value	Regularization	Value
Backbone (video)	MAE-Face	Drop path rate	0.1
Backbone (audio)	AudioMAE	Embedding dimension	512
Video patch size	16	Color jitter	0.4
Adapter dimension	64	Random erase prob	0.25

**Table 2:** MFAdapter and fusion module architecture details.

Component	Parameter	Value
MFAdapter	Bottleneck dimension	64
	Projection (in/out)	768→64, 64→768
	Mamba $d_{\mathrm{state}}$	16
	Mamba $d_{\mathrm{conv}}$ (kernel)	4
	Mamba expand factor	2
	SSM inner dimension	2×64=128
	$d_{t}$ (dt_rank)	⌈64/16⌉=4
	Activation	SiLU
	Trainable params (per layer)	∼197.8 K
	Insertion depth	All 12 Transformer blocks

**Table 3:** WAR/UAR/WF1 (%). Each fold: mean ± std over 3 seeds; bottom rows: overall mean ± std (n=15) and 95% CI of the mean. DFEW: 76.62/65.25/76.71; MAFW: 58.70/43.66/60.70.

Dataset	Fold	WAR (%)	UAR (%)	WF1 (%)
DFEW	Fold 1	76.80±0.45	64.14±0.54	77.43±0.53
	Fold 2	74.57±0.07	63.46±0.11	74.16±0.07
	Fold 3	76.94±0.62	66.03±0.90	77.34±0.77
	Fold 4	76.64±0.20	66.08±0.39	76.36±0.11
	Fold 5	77.81±0.81	65.89±0.89	78.25±0.77
	**Mean ± std**	76.55±1.20	65.12±1.23	76.71±1.57
	95% CI of the mean	[75.89,77.21]	[64.44,65.80]	[75.84,77.57]
MAFW	Fold 1	49.38±0.14	35.92±0.46	51.32±0.19
	Fold 2	54.92±0.08	39.93±0.82	58.08±0.79
	Fold 3	62.65±0.86	47.73±0.68	64.26±0.77
	Fold 4	64.13±0.45	48.47±1.19	66.54±0.16
	Fold 5	60.99±0.52	46.03±0.81	63.29±0.60
	**Mean ± std**	58.41±5.71	43.61±5.10	60.70±5.66
	95% CI of the mean	[55.26,61.57]	[40.79,46.44]	[57.57,63.83]

**Table 4:** Comparison of state-of-the-art methods on DFEW (7-class) and MAFW (11-class) datasets (UAR/WAR, %). Total: total parameters; Train.: trainable parameters (frozen backbones excluded for PEFT methods marked with ^†^). The best results are shown in bold, and the second-best are underlined.

Method	Params (M)		DFEW		MAFW	
	Total	Train.	UAR	WAR	UAR	WAR
Wav2Vec2.0 [49]	95	95	36.15	43.05	21.59	29.69
HuBERT [50]	95	95	35.98	43.24	25.00	32.60
WavLM-Plus [51]	95	95	37.78	44.64	26.33	34.07
C3D [52]	78	78	42.74	53.54	31.17	42.25
R(2+1)D-18 [53]	-	-	42.79	53.22	-	-
C3D+LSTM [8]	-	-	55.35	65.17	30.47	44.15
ResNet18+LSTM [11]	-	-	51.32	63.85	28.08	39.38
T-MEP [19]	61	61	57.16	68.85	37.17	51.15
AMH [54]	-	-	54.48	66.51	32.98	48.83
IAL [25]	19	19	55.71	69.24	-	-
DFER-CLIP [14]	90	90	59.61	71.25	38.89	52.55
FE-Adapter ^†^ [55]	–	6.6	60.89	73.67	39.41	55.02
MAE-DFER [10]	85	85	63.41	74.43	41.62	54.31
HiCMAE [15]	81	81	63.76	75.01	42.65	56.17
MMA-DFER ^†^ [33]	-	7.5	**67.01**	**77.51**	**44.11**	58.52
**Our Method**	175.6	4.7	65.25	76.62	43.66	**58.70**

**Table 5:** Per-class and overall (UAR/WAR, %) results on DFEW. *: with oversampling. Params: Total/Train. (M); for PEFT methods marked ^†^, Train. counts only updated parameters. Best in bold, second best underlined.

Methods	Total/Train. (M)	Happy	Sad	Neutral	Angry	Surprise	Disgust	Fear	UAR	WAR
C3D [52]	78/78	75.17	39.49	55.11	62.49	45.00	1.38	20.51	42.74	53.54
3D ResNet-18 [56]	33/33	76.32	50.21	64.18	62.85	47.52	0.00	24.56	42.79	53.22
Former-DFER [11]	18/18	84.05	62.57	67.52	70.03	56.43	3.45	31.78	53.69	65.70
CEFLNet [24]	13/13	-	-	-	-	-	-	-	51.14	65.35
EST [9]	43/43	-	-	-	-	-	-	-	53.94	65.85
NR-DFERNet [57]	-/-	88.47	64.84	70.03	75.09	61.60	0.00	19.43	54.21	68.19
STT [58]	-/-	87.36	67.90	64.97	71.24	53.10	3.49	34.04	54.58	66.65
DFER-CLIP [14]	90/90	-	-	-	-	-	-	-	59.61	71.25
IAL [25]	19/19	87.95	67.21	70.10	76.06	62.22	0.00	26.44	55.71	69.24
M3DFEL [59]	-/-	89.59	68.38	67.88	74.24	59.69	0.00	31.64	56.10	69.25
AEN [60]	-/-	89.24	69.38	70.67	72.08	59.07	4.17	26.44	56.66	69.37
CDGT [61]	16/16	89.90	70.86	63.99	73.77	58.88	17.24	39.47	59.16	70.07
MSSL [62]	-/-	87.80	69.60	64.60	71.20	54.40	14.26	38.00	60.09	69.91
MIDAS [27]	-/-	87.40	67.34	58.64	68.06	59.65	28.69	44.50	57.45	69.16
MAE-DFER [10]	85/85	92.92	77.46	74.56	76.94	60.99	18.62	42.34	63.41	74.43
SVFAP [63]	78/78	93.13	76.98	72.31	77.54	**65.42**	15.17	39.25	62.83	74.27
S2D ^†^ [64]	-/9	93.87	**83.25**	**75.31**	**84.19**	64.33	0.00	37.07	62.57	75.98
**Our Method**	175.6/4.7	94.35	79.35	74.56	80.21	63.65	13.10	57.63	65.25	**76.62**
Our Method *	175.6/4.7	**94.44**	79.41	69.28	76.21	60.10	**37.93**	**57.77**	**67.87**	75.04

**Table 6:** UAR and WAR on DFEW for audio-only, video-only, and audio-visual inputs with/without adapters.

Modality	Adapter	UAR	WAR
Audio-only	W/O Adapter	32.72	42.97
Audio-only	Unimodal Adapter	36.58	46.01
Video-only	W/O Adapter	52.70	67.11
Video-only	Unimodal Adapter	57.90	71.17
Audio-Visual	W/O Adapter	54.96	68.77
Audio-Visual	MFAdapter	65.25	76.62

**Table 7:** Controlled audio ablations on DFEW. muted: silent waveform; permuted: 4-segment shuffled waveform; capacity_control: constant-token branch matching MFAdapter parameters; train_muted/test_time_mute: audio silenced only in training or inference. Main and Video-only rows from Table 6.

Condition	UAR (%)	WAR (%)
Video-only + Unimodal Adapter	57.90	71.17
capacity_control (constant-token branch)	59.71	73.17
train_muted	59.19	72.77
muted	62.42	75.65
permuted	62.97	76.12
test_time_mute	63.54	75.23
Our Method	65.25	76.62

**Table 8:** Comparison of Separate Adapters and MFAdapter on the DFEW dataset.

Modality	Adapter	UAR	WAR
Audio-Visual	Separate Adapters	59.68	73.22
Audio-Visual	MFAdapter	65.25	76.62

**Table 9:** Effect of adapter number and position on DFEW. Train. denotes trainable parameters; the total model size remains 175.6 M for all settings.

Layers	Train. (M)	UAR	WAR
1	2.2	60.73	73.69
6	2.2	61.08	73.81
12	2.2	59.04	71.29
1–4	3.0	61.16	73.67
5–8	3.0	61.41	75.27
9–12	3.0	62.16	75.69
1–12	4.7	65.25	76.62

**Table 10:** Comparison of direct concatenation and FFA on the DFEW dataset.

Fusion Strategy	Adapter	UAR	WAR
Concat	MFAdapter	61.30	74.84
FFA	MFAdapter	65.25	76.62

**Table 11:** Ablation of MFAdapter fusion operators on DFEW. Train. denotes trainable adapter parameters.

Type	Train. (M)	UAR	WAR
Identity	4.2	60.76	72.96
MLP	4.3	63.35	74.92
CrossAttn	4.6	61.06	74.07
LoRA	4.2	63.93	75.01
MFAdapter	4.7	65.25	76.62

**Table 12:** Effect of downsample dimension $D_{\mathrm{down}}$ on DFEW under identical training settings. Train. denotes trainable parameters; Total varies with $D_{\mathrm{down}}$ because the adapter width changes, while frozen backbones remain included at inference.

$D_{down}$	Train. (M)	UAR	WAR
32	3.3	63.40	75.50
64	4.7	65.25	76.62
128	8.1	65.54	76.85
256	16.7	64.82	76.33

**Table 13:** Computational efficiency of full fine-tuning vs. MFAdapter on DFEW. Memory and latency are measured on a single NVIDIA A100 GPU in FP32 at batch size 8 with torch.cuda.max_memory_allocated() under model.eval() + torch.no_grad() for inference and standard training for the train column (including gradients and AdamW state). Both runs use the same 175.6 M-parameter architecture; only the 171.0 M frozen-backbone subset differs in trainable scope. For inference, both measurements run under model.eval() + torch.no_grad(), which disables the autograd graph entirely; consequently, param.requires_grad does not alter activation-caching and gradient checkpointing (a training-time technique) is not triggered.

Method	Params (M)		Memory (GB)		Latency (ms)	
	Total	Train.	Train.	Infer.	Train.	Infer.
Full fine-tuning	175.6	175.6	22.1	2.42	–	55.28
MFAdapter (PEFT)	175.6	4.58	14.1	1.76	–	55.24

**Table 14:** Full fine-tuning vs. adapter-based PEFT on DFEW (same settings). Total: all parameters loaded at inference; Train.: parameters updated during optimization.

Method	Total (M)	Train. (M)	UAR	WAR
Full fine-tuning	175.6	175.6	63.39	77.53
MFAdapter (PEFT)	175.6	4.7	65.25	76.62

## Data Availability

The DFEW and MAFW datasets used in this study are publicly available under controlled-access conditions. They are not readily downloadable because their data providers require users to submit formal access applications and agree to the applicable terms of use before access is granted. Furthermore, the licensing terms of these datasets do not permit the authors to redistribute the original data to third parties. The DFEW dataset can be requested through its official application page at https://dfew-dataset.github.io/download.html (accessed on 31 October 2023). Access requests should be directed to Xingxun Jiang (jiangxingxun@seu.edu.cn) and Yuan Zong (xhzongyuan@seu.edu.cn), as specified by the data provider. The MAFW dataset can be requested through its official application page at https://mafw-database.github.io/MAFW/ (accessed on 14 October 2024). Applicants are required to complete and submit the End-User License Agreement to the dataset custodian at 1202411179@cug.edu.cn for review and approval. No new datasets were created in this study.
